# A Combined Approach to Assess the Microbial Contamination of the Archimedes Palimpsest

**DOI:** 10.1007/s00248-014-0481-7

**Published:** 2014-08-19

**Authors:** Guadalupe Piñar, Katja Sterflinger, Jörg Ettenauer, Abigail Quandt, Flavia Pinzari

**Affiliations:** 1 Institute of Applied Microbiology, Department of Biotechnology, University of Natural Resources and Life Sciences, Muthgasse 11, 1190 Vienna, Austria; 2 The Walters Art Museum, Book and Paper Conservation, 600 North Charles St., Baltimore, MD 21201 USA; 3 Laboratorio di Biologia, Ministero per i Beni e le Attivita Culturali, Istituto Centrale per il Restauro e la Conservazione del Patrimonio Archivistico e Librario (ICRCPAL), Via Milano 76, 00184 Rome, Italy; 4 Present Address: Consiglio per la Ricerca e la sperimentazione in Agricoltura, Centro di ricerca per lo studio delle relazioni tra pianta e suolo, Via della Navicella 2-4, 00184 Rome, Italy

## Abstract

A combined approach, using molecular and microscopic techniques, was used to identify the microbiota associated with the Archimedes Palimpsest, an unusual parchment manuscript. SEM analyses revealed the microbial damage to the collagen fibers and the presence of characteristic cell chains typical of filamentous bacteria and fungal spores. Molecular analysis confirmed a homogeneous bacterial community colonizing the manuscript. The phyla *Proteobacteria* and *Actinobacteria* were associated with this ancient parchment; the sequences were most related to uncultured clones detected in the human skin microbiome and in ephitelium, and to cultivated species of the genera *Acinetobacter* and *Nocardiopsis*. Nevertheless, a great variation was observed among the different sampled areas indicating fungal diversity. *Blumeria* spp. dominated in the healthy areas of the parchment while degraded areas showed disparate fungal communities, with dominant members of the genera *Mucor* and *Cladosporium*. In addition, the quantification of the β-actin gene by real-time PCR analyses (qPCR) revealed a higher fungal abundance on degraded areas than on the healthy ones.

## Introduction

The transmission of ancient texts through the ages appears to be an almost miraculous event from both the microbiological and cultural point of view. Most palimpsests known to modern scholars are made of parchment, a material that rose in popularity as a writing support in Western Europe after the sixth century. Parchment is prepared from animal hides [1, 2], and, although it is far more durable than paper or papyrus, it is still highly degradable by microorganisms like filamentous bacteria and proteolytic fungi. In the course of ten or more centuries, the probability that an organic material will be attacked by some saprophytic organism is very high. The medieval codices that have survived must have faced the most biodiverse communities of organisms and been exposed to fires, floods, dust, and contamination of all kinds for centuries. In theory, the pages of an ancient manuscript bear the scars of past events in the form of damages, but also in the DNA of all the microorganisms that have come and gone during the natural decomposition of the organic substrate. Like archeological sites and their contents, ancient books are unique records of our history, not only in the texts and illuminations that they contain but also in their material being; a sort of archive of past events that once restored, are gone forever. This is not to say that ancient codices should not be repaired and restored, but that a modern approach should take into account possible future scientific developments in “microbial-archeology” and the study of past events that can tell something more about these objects.

Examples of microbiological archeology and studies focusing on ancient biological damage of cultural heritage materials have recently been published [3–7]. In this context, techniques based on molecular biology are becoming more informative and capable of disclosing valuable information about both recent and past microbial contamination, environmental conditions, and particular events to which the objects were exposed [8–10]. In the case of the Archimedes Palimpsest, the restoration was aimed at being minimally invasive and, as a result, information on the materiality of the object remains accessible to future generations of scientists. The study of the causes and effects of degradation phenomena observed on valuable objects, coupling different analytical techniques, is an approach endorsed by all the scientists that works on cultural heritage conservation, but the biological component is very often the most complex to investigate because it itself depends on many variables, and only multidisciplinary studies can lead to convincing results [11–14].

**Fig. 1:**
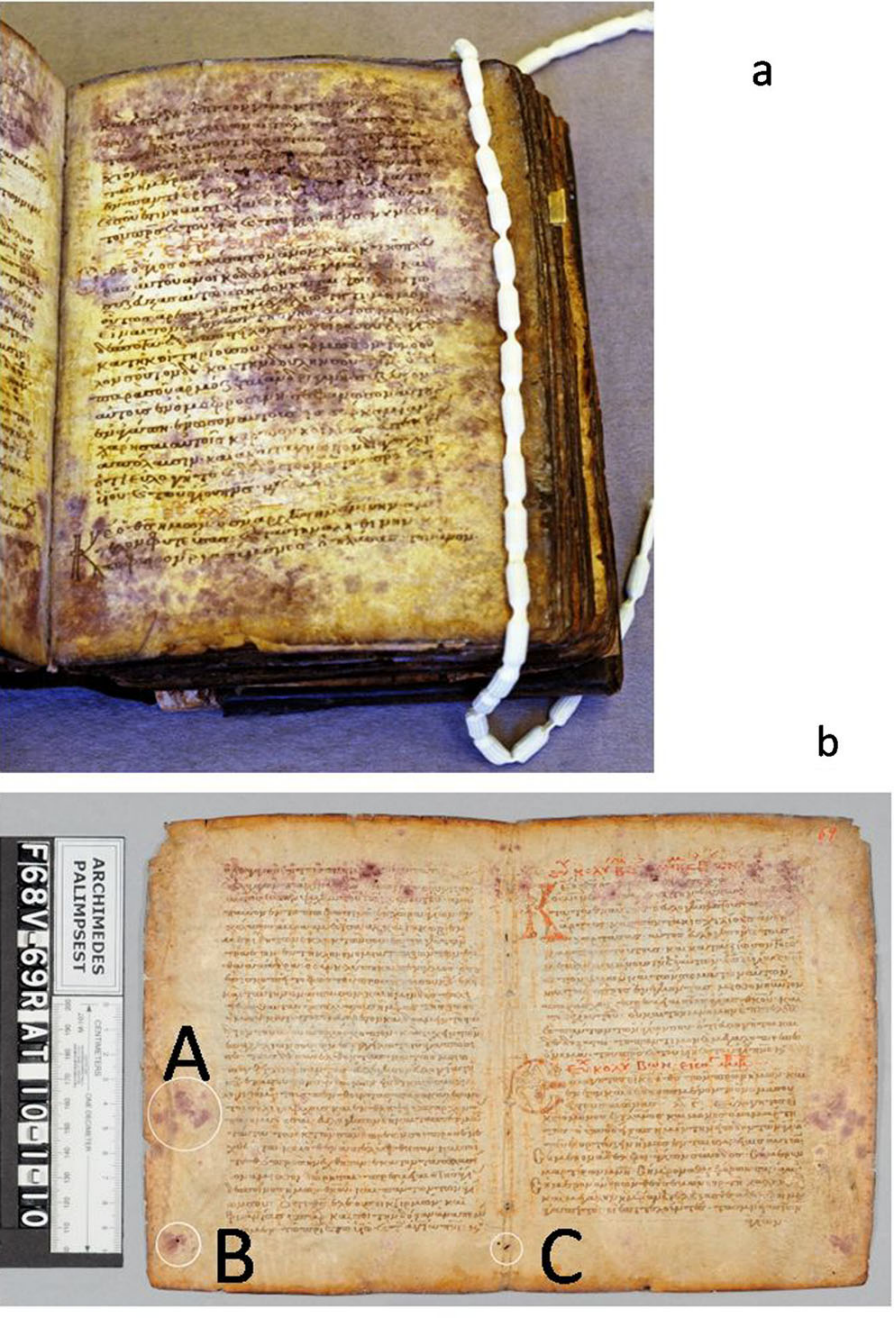
**a** The Archimedes Palimpsest before treatment, showing intense *purple stains* and large areas of degraded parchment. **b** The picture shows the folios 68v-69r of the manuscript before the conservation treatment; the three sample sites swab AP1_ f.68v, taken from the *lower left corner* and swab AP3_ f.68v, from the *center of the outer margin* (locations *A* and *B*). The third swab sample was collected from a healthy area near a sewing hole along the centerfold that showed no visible damage: sample AP2_ f.68v (location *C*) (Images taken at the Walters Art Museum are copyright of the owner of the Archimedes Palimpsest)

The Archimedes Palimpsest is a thirteenth century parchment codex that was made from recycled folios of six different Greek texts, including the earliest surviving copy of Archimedes treatises that dates to the tenth century. The palimpsest is the unique source for two of Archimedes treatises, “The Method” and “Stomachion”, and the unique source for the treatise “On Floating Bodies” in the original Greek. Discovered in 1906 by J.L. Heiberg, the palimpsest played a prominent role in his 1910–1915 edition of the works of Archimedes, upon which all subsequent work on Archimedes has been based. The palimpsest was in private hands throughout much of the twentieth century and was sold at auction to a private collector on the 29th of October 1998. The new owner deposited the manuscript at The Walters Art Museum in Baltimore, Maryland in January of 1999 for the purpose of conservation, imaging, and transcription of the erased Greek texts. Work on the Palimpsest, funded by the owner, has been ongoing ever since. The disbinding of the manuscript started in February 2000, and finished in November 2004. Thereafter, each leaf was imaged, and Quandt [11] undertook further work to stabilize and preserve them for the future. Finally, each leaf was encased in a double-sided frame and in this way, the leaves were shown in a dedicated exhibition (“Lost and Found: The Secrets of Archimedes”, The Walters Oct. 16, 2011, Baltimore) and then, finally returned to its anonymous owner. A dedicated climate control system will be incorporated into the custom storage unit that is being designed by the current owner to ensure the preservation of the Archimedes Palimpsest for future generations [11]. Scientific research relating to the ideal long-term storage conditions for parchments demonstrated that the standard 50–65 % relative humidity that exists for the conservation of artifacts in many American and European collections can encourage the further deterioration of ancient, degraded collagen. Recommendations were therefore made to store the Archimedes Palimpsest at lower moisture levels of 30 % relative humidity with a cyclic variation of ±5 % relative humidity [15]. When first examined by the Walters senior conservator Quandt, the Archimedes Palimpsest exhibited significant damage (Fig. 1a). “The edges of the book were gelatinized and charred black from exposure to a fire, and the parchment was heavily distorted. The leaves were covered with various stains and a thick layer of grime; at the outer edges were deposits of a gritty substance that resembled sand. The inks of the prayer text were badly cracked and flaking, and ink corrosion was present in both layers of writing. Every folio was disfigured by dark purple spots. Many leaves were like lace, possibly perforated by fungi that have eaten through the parchment” [11].

The treatment of this manuscript was extremely difficult, as the conservator had to deal with the multiple effects of fire, water, and mold on the recycled parchment, which bore two layers of writing on each side. In the first 3 years of the project, scientists from the Canadian Conservation Institute helped to assess the condition of the inks and the parchment substrate. During this first survey, culturing and biochemical analyses were addressed to define the activity of the microbial attack [16]. While the microorganisms that had attacked the manuscript were no longer active, they had heavily stained and perforated the parchment, making it very weak and subject to further damage. In 2010, an opportunity arose to obtain more specific information about the microorganisms that had attacked the parchment and left it in such a deteriorated condition. As with all testing done on the palimpsest throughout the course of the project, a proposal for the work was made to the owner, who gave his approval for microscopic samples to be taken in discreet areas that were blank on both sides. These valuable samples have been used in this study to investigate the microbial community associated with this unusual manuscript by means of microbial, microscopic, and molecular investigations. Nucleic acid-based strategies targeting rRNA-encoding regions were selected for essays studying the community structure of fungi and bacteria. In addition, quantitative real-time polymerase chain reaction (PCR) of the fungal β-actin gene was conducted in order to evaluate and compare the current fungal abundance on both healthy and damaged parts of the parchment.

## Materials and Methods

### Case Study

Samples were taken from two folios by Walters’ conservation scientist Glenn Gates and conservator Abigail Quandt, who followed standard protocols by using sterile procedures for the sampling. Samples from the surface of three sites were taken with sterile cotton swabs. Two of the samples were collected from areas showing purple mold stains and degraded parchment: sample AP1_ f.68v, taken from the lower left corner and AP3_ f.68v, from the center of the outer margin (locations A and B in Fig. 1b). The third swab sample was collected from a healthy area near a sewing hole along the centerfold that showed no visible damage: sample AP2_ f.68v (location C in Fig. 1b). These swab samples were used for molecular assays.

**Fig. 2:**
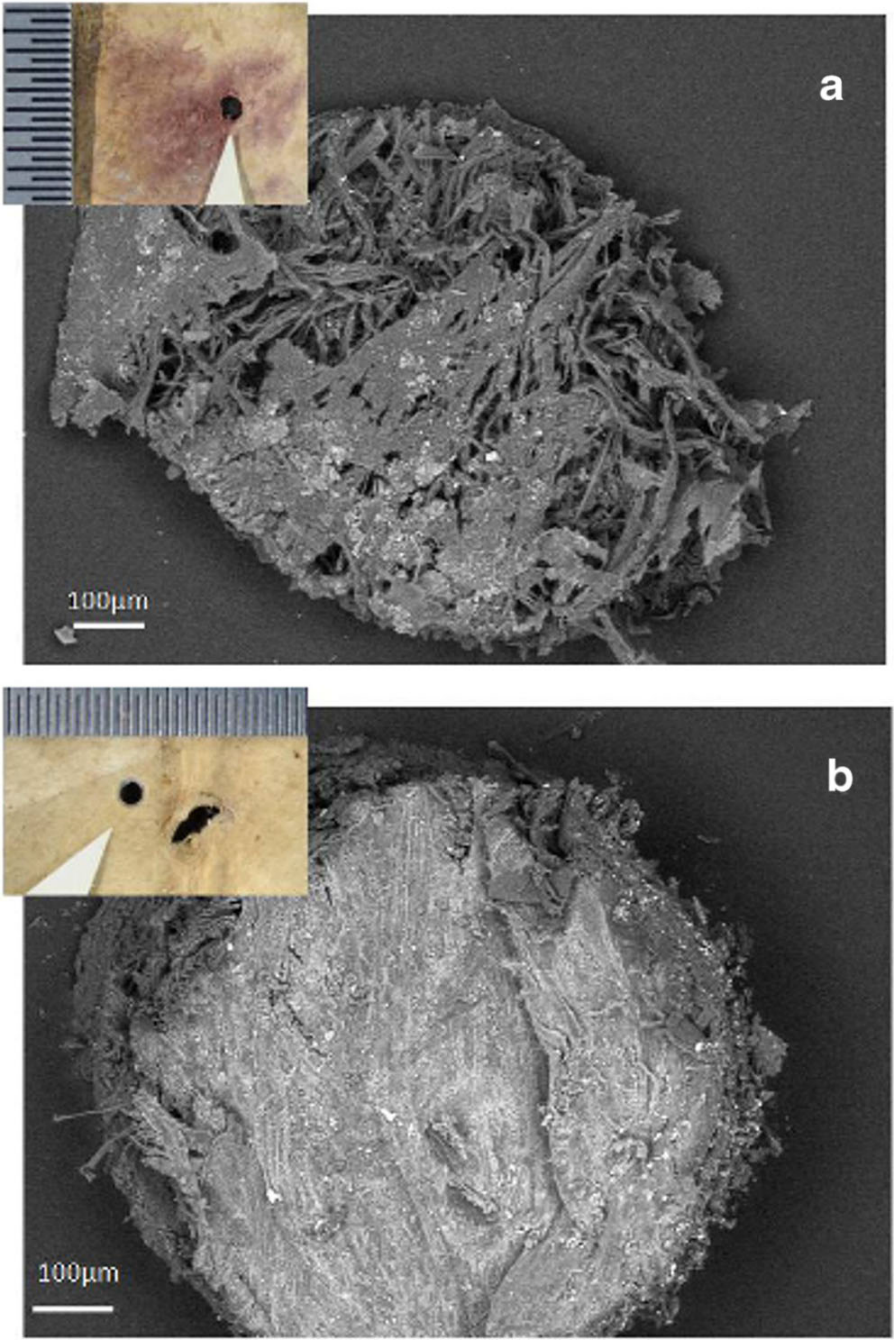
Core samples taken from purple stained and degraded (**a**) and healthy/control areas (**b**) of folio 68v. **a** SEM low magnification image of the core taken from a stained and degraded area (location *B* in **b**), and obtained at variable pressure (50 Pa) with a backscattered electron detector on uncoated material. **b** SEM low magnification image of the core obtained from a healthy/control area (location *C* in Fig. 1b). *Inset* in both images are photomicrographs taken at the Walters Art Museum of the tiny holes left in the parchment after the core samples were removed (Images taken at the Walters Art Museum are copyright of the owner of the Archimedes Palimpsest)

A total of three core samples were taken from blank areas of two folios with a hypodermic needle, which left a 1-mm diameter hole in the parchment. One core sample came from a stained and degraded area (Figs. 1b and 2a) and one from a healthy area (Figs. 1b and 2b) of the same folio, while a second stained and degraded core sample was taken from another folio. Photomicrographs were taken of all locations before and after sampling, and notes were made in the conservation record for each folio. The core samples were used for low-vacuum observations with a variable pressure scanning electron microscopy (SEM) coupled with backscattered electron diffraction (BSD), and in high-vacuum SEM observations, after metallization, to examine the spore morphology and the remnants of fungal and bacterial attack.

### Molecular Analyses

#### DNA Extraction

DNA extraction was performed as described by Piñar et al. [7] directly from the three cotton swabs using a method previously described by Sert and Sterflinger [17] with the following modifications: cotton swabs together with 500 ml lysing buffer were added to the tubes of the lysing matrix A (MP Biomedicals). The mixture was shaken in a cell disrupter (Thermo Savant FastPrep, FP120, Holbrook, USA) at full speed for 40 s and incubated for 1 h at 65 °C. Afterwards, the mixture was shaken again at full speed for 40 s and then centrifuged for 10 min at 10,000*×g*. The supernatant was transferred to a new Eppendorf tube and an approximately equal volume of chloroform/isoamyl alcohol [24:1 ratio] was added, mixed thoroughly, and centrifuged for 5 min in a microcentrifuge. The supernatant was transferred to a new Eppendorf tube and further purified using the QIAamp Viral RNA mini kit (Qiagen, Hilden, Germany) following the instructions of the manufacturer. The final elution step was repeated twice with 100 ml of 80 °C preheated ddH2O (Sigma Aldrich, St. Louis, MO, USA). The purified DNA was used directly for PCR amplification.

After the DNA extraction and purification, the concentration and quality of the DNA was assessed using a NanoDrop® ND-1000 Spectrophotometer (peqLab Biotechnologie GmbH, Linz, Austria). The analyses were performed according to the manufacturer’s protocol and the extracted DNA was analyzed in duplicate. The purified DNA was used directly for PCR amplification.

#### PCR Amplification of Extracted DNA

For all PCR reactions, 2× PCR Master Mix from Promega (Vienna, Austria) [50 unit ml^−1^ of *Taq* DNA polymerase supplied in an appropriate reaction buffer (pH 8.5), 400 μM dATP, 400 μM dGTP, 400 μM dCTP, 400 μM dTTP, and 3 mM MgCl2] was diluted to 1*×*, and 12.5 pmol μl^−1^ of each primer (stock: 50 pmol μl^−1^, VBC-Biotech, Austria) were added. In a total volume of 25 μl, 400 μg ml^−1^ BSA (stock: 20 mg ml^−1^; Roche, Diagnostics Gmbh, Germany) and 2.5 μl DNA template were added. PCR was performed in a MJ Research PTC-200 Peltier Thermal Cycler.

For the analysis of fungal sequences, fragments of 450–600 bp in size corresponding to the ITS1, the ITS2 region, and the adjacent 5.8S rRNA gene were amplified with the primer pair ITS1 and ITS4 [18]. For denaturing gradient gel electrophoresis (DGGE) analysis, a nested PCR was performed with the PCR product of the first round as template DNA using the primers ITS1GC with a 37-base GC-clamp attached to the 5′ end [19] and ITS2. All reactions were carried out as described by Michaelsen et al. [9].

For the amplification of bacterial 16S rRNA gene sequences, DNA was amplified with the primer pair 341f/985r [19, 20]. For DGGE analysis, 200 bp fragments of the 16S rDNA were amplified with a nested PCR using the eubacterial specific primer 341f-GC with a 40-bp GC clamp added to its 5′ end [19] and the universal consensus primer 518r [21]. PCR conditions were as described by Schabereiter-Gurtner et al. [8].

#### Denaturing Gradient Gel Electrophoresis

**Fig. 3:**
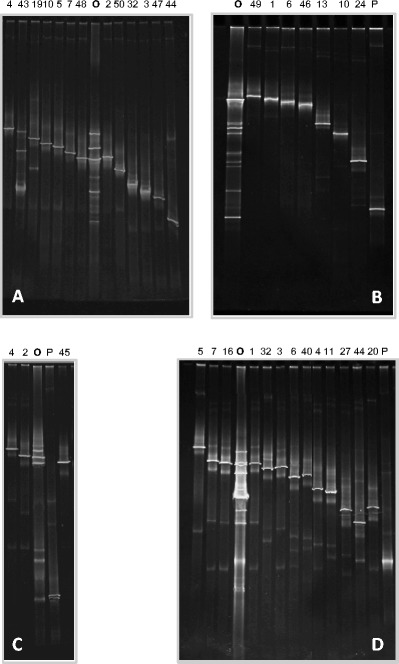
PCR-DGGE-fingerprints derived from **a** the bacterial community colonizing all three pooled swab samples and **b** the fungal communities colonizing the swab sample AP1, **c** the swab sample AP2, and **d** the swab sample AP3 of the Archimedes Palimpsest, as well as the PCR-DGGE profiles of sequenced clones containing 16S rDNA bacterial fragments (**a**) and ITS regions (**b**–**d**) producing PCR products with different motility behavior. *O* Original fingerprint derived from swab samples. *P* clones matching plant DNA. The *numbers of the lanes* indicate the number of the corresponding sequenced clones quoted in Table 1 and 2. The linear chemical gradient of denaturants used was 25–60 % for bacteria and 20–50 % for fungi

DGGE was performed as previously described [19] using a D-Code system (Bio-Rad) in ×0.5 TAE (20 mM Tris, 10 mM acetate, 0.5 mM Na_2_ EDTA; pH7.8 with 8 % (*w v* ^−1^ acrylamide). Gels were run at a constant temperature of 60 °C with a voltage of 200 V during 3.5 h for bacteria, and 5 h for fungal fingerprints. The linear chemical gradient of denaturants used in this study [100 % denaturing solution contains 7 M urea and 40 % (*v v* ^−1^) formamide] are indicated in the legend of Fig. 3.

After completion of electrophoresis, gels were stained in a 1 μg ml^−1^ ethidium bromide solution [stock: 10 mg ml^−1^] for 20 min and afterwards visualized by a UVP documentation system (BioRad Transilluminator, Universal Hood; Mitsubishi P93D-printer).

#### Creation of Clone Libraries and Sequence Analysis

To obtain a detailed phylogenetic identification of the microbial community members, clone libraries containing either ITS fungal regions (fungal community) or 16S rRNA gene fragments (bacterial communities) were carried out. For the construction of clone libraries, 2 × 3 μl DNA templates of each sample were amplified in 2 × 50 μl reaction volumes using the following primer pair combinations: for fungal clone libraries, the DNA template was amplified using the primers ITS1/ITS4, as mentioned above. For bacterial clone libraries, the primer pair 341f/985r was used as mentioned above. The PCR products were purified using the QIAquick PCR Purification Kit Protocol (Qiagen, Hilden, Germany) and resuspended in ddH2O water.

Purified PCR products were ligated into the pGEM-T easy Vector system (Promega, Vienna, Austria) following the instructions of the manufacturer. The ligation products were transformed into One shot TOP10 cells (Invitrogen). These cells allow the identification of recombinants (white colonies) on an indicator LB medium containing ampicillin (100 μg ml^−1^), streptomycin (25 μg ml^−1^), and X-Gal (5-bromo-4-chloro-3-indolyl-ß-1-galactopyranoside; 0.1 mM) [22].

Fifty clones per each clone library were screened in a DGGE gel as described by Schabereiter-Gurtner et al. [8]. Selected clones were externally sequenced by Sanger sequencing with a fleet of 16 ABI 3730xl (GATC Biotech, Germany). Comparative sequence analysis was performed by comparing pair-wise insert sequences with those available in the public online database National Center for Biotechnology Information (NCBI) using the Basic Local Alignment Search Tool (BLAST) search program [23] and in addition, the most similar sequences were searched using the Ribosomal Database Project (RDP-II) applying the SeqMatch tool [24]. The resulting sequences of the bacterial and fungal clones have been deposited at the GenBank: genetic sequence database at the National Center for Biotechnical Information.

#### Quantitative Real-Time PCR

Quantitative real-time PCR was performed in a BioRad CFX96™ real-time PCR by using the SensiMix Plus™ SYBR-Kit (Bioline). Each 20-μl reaction contained 10 μl SensiMix-Plus, 1 μl 50 mM MgCl_2_ (final conc. 2.5 mM), 0.25 μl of a 10 pmol/μl primer solution using the β-actin primers ACT 512-F (5′ ATG TGC AAG GCC GGT TTC GC 3′) and ACT 783-R (5′ TAC GAG TCC TTC TGG CCC AT 3′) [25], 6.5 μl H_2_O, and 2 μl of DNA template. The amplification conditions were 95 °C for 10 min and then 40 cycles of 95 °C 15 s, 61 °C 20 s, and 72 °C 15 s. Fluorescence measurements were made at the end of each annealing cycle and an additional measuring point at 80 °C (for 1 s) to detect the formation of primer dimers during amplification. A melt curve analysis was made by raising the temperature from 65 to 95 °C in 0.5 °C steps for 5 s each.

To enable the quantification of PCR products, standard curves based on threshold cycles were produced by re-amplifying tenfold dilution series of PCR products from genomic DNA. An aliquot of each dilution (0.035 fg–0.035 ng, equivalent to 1 × 10^2^–1 × 10^8^ β-actin copies) in three replicates were used as templates in real-time PCR. The DNA standards were generated with the β-actin primers, mentioned above, with the following PCR program: 95 °C for 3 min and the 30 cycles of 95 °C 30 s, 55 °C 30 s, 72 °C 30 s, and a final elongation step at 72 °C for 1 min. PCR was done with a BioRad C1000 thermal cycler using the PCR Master Mix (Promega, Mannheim, Germany) [50 units/ml of TaqDNA Polymerase in a supplied reaction buffer (pH 8.5), 400 μM dATP, 400 μM dGTP, 400 μM dCTP, 400 μM dTTP, 3 mM MgCl_2_]. Each 100 μl reaction contained 50 μl 2× PCR Master Mix, each 1 μl of forward and reverse primer (stock, 10 pmol/μl), 43 μl ultrapure water and 5 μl template of genomic DNA of *Aspergillus niger*. The PCR products were cleaned using the QIAquick PCR Purification kit (QIAGEN) and checked for purity on agarose gels and by sequence analysis with database comparison. Concentration of the PCR product was measured spectrophotometrically at 260 nm with a NanoDrop® ND-1000. The resulting PCR products were used to construct standard curves for absolute quantification. The numbers of copies in the standards were calculated using the formula from Le Calvez [26] and various online tools, like from the URI Genomics & Sequencing Center (http://cels.uri.edu/gsc/cndna.html). Standard curves were automatically generated by the BioRad Precision Melt Analysis™ software.

### SEM-EDX Technique and Statistical Comparisons

The parchment core samples taken from both stained and degraded (Fig. 2a) and healthy areas (Fig. 2b) were analyzed using a variable pressure SEM instrument (EVO50, Carl-Zeiss Electron Microscopy Group) equipped with a detector for backscattered electron diffraction (BSD). A chemical characterization of the inorganic constituents of the samples was performed by means of electron dispersive spectroscopy (EDS). After having observed all three samples with SEM in variable pressure mode, at 20 keV, with the backscattered electron detector (BSD), some of the samples were covered with gold with a Baltec Sputter Coater for further analysis in high-vacuum mode. The sputtering was performed under an argon gas flow, at 50 mm working distance with 0.05 mbar of pressure and a current of 40 mA, for 60 s to obtain a film of gold of about 15 nm. Reference elemental intensities acquired from pure compounds (standards) are commonly utilized for calibrating SEM-EDX systems. In the case study described in this paper, conventional ZAF correction (13) integrated into an Oxford INCA 250 microanalysis package was applied to the spectrum dataset (Oxford Instruments).

The EDS measurements were taken at several points across the surface of both the hair and flesh sides of the three core samples. Each measurement was then repeated in at least three areas to obtain up to 60 repeated measurements for each sample on each side. The data obtained was used for a series of comparisons aimed at evaluating the relationships between the variables and the significance of the differences between the damaged and healthy parchment cores. One-way analysis of the variance (ANOVA) was applied when comparing the different sides of the samples, and the significance of the differences was tested at 95 % confidence. The ANOVA model used is “balanced” because the number of observations within each category was the same. ANOVA was followed by a post hoc analysis using Tukey’s honestly significant difference (HSD) *t* test [27]. PCA was used to study and visualize the correlations between all the variables (chemical elements) [28]. The first few principal components (PCs) resulting from PCA are generally utilized to analyze the common features among samples and their grouping: samples characterized by similar elemental signatures tend, in fact, to aggregate in the score plot of the first two or three components. Samples characterized by similar elemental composition are thus grouped in the same region of the score plot related to the first two PCs, whereas samples with different elemental features are clustered in other parts of this space.

## Results and Discussion

### DNA Extraction, Amplification, and DGGE Fingerprinting

DNA could be extracted from the three cotton swabs yielding a concentration of 70.35–74.50 ng DNA μl^−1^ and was further amplified by PCR with primers targeting the 16S rDNA of bacteria as well as the ITS regions of fungi. PCR analysis using bacterial- and fungal-specific primers showed positive results. The bacterial 16S rDNA and the fungal ITS-amplified fragments were further analyzed using DGGE fingerprints. This technique enabled an estimation of the most abundant organisms inhabiting this unusual parchment object, their comparison among the different sampled areas, as well as the screening of the cloned sequences. The DGGE profiles obtained from the swab samples as well as from the corresponding sequenced clones are shown in Fig. 3a (bacteria) and in Fig. 3b–d (fungi). DGGE fingerprints derived from bacterial 16S rDNA amplified from the three independent cotton swabs were shown to be identical (data not shown). Therefore, aliquots of these DNAs were pooled for the construction of a single bacterial clone library (Fig. 3a). On the contrary, DGGE fingerprints derived from ITS fungal regions amplified from the three cotton swabs showed relevant differences (Fig. 3b–d) so all of them were further investigated by cloning and sequencing.

### Phylogenetic Identification of the Microbial Communities Colonizing the Parchment

To accomplish phylogenetic identification of the bacterial and fungal communities inhabiting the parchment folios of the Archimedes Palimpsest, clone libraries containing the 16S rDNA (one clone library) or the ITS fragments (three clone libraries) were generated. Clones were screened by DGGE and those displaying different fingerprints were grouped. Finally, one representative of each group was selected for sequencing (Fig. 3).

#### Bacterial Community

Comparative sequence analysis of the selected bacterial clones was performed by using the public online database NCBI using the BLAST search program, as mentioned in the “Materials and Methods” section. Results derived from BLAST search yielded, in most cases, high similarity ranges (99–94 % similarity) with uncultured clones, being not always possible to get any affiliation with validated species names (Table 1). Therefore, additionally, the most similar sequences were searched in the RDP-II database by using the SeqMatch tool [24], with the type strain option for sequence homology search, in order to get information about the more exact phylogenetic positions of the uncultured clones. Table 1 contains both data, retrieved from BLAST (closest relative) and RDPII (validated species as closest cultivated relative) searches.

**Table 1:** Phylogenetic affinities of partial 16S rRNA coding sequences retrieved from swab samples of the Archimedes Palimpsest

Phylum	Clones (%) and selected clone	Closest identified phylogenetic relatives [EMBL accession numbers] and validated species as closest relatives [RDP accession numbers]	Similarity (%)	Acc. Nr.
*Proteobacteria* (Alpha-class) (6 %)	2 % AP-K3	Uncultured bacterium clones 16S ribosomal RNA gene, partial sequence [GQ009750; GQ009436] from the human skin microbiome. *Caedibacter caryophilus* ^a^ (T); 221. [X71837].	99.0 94.4	KF983492
	2 % AP-K32	Uncultured bacterium clones 16S ribosomal RNA gene, partial sequence [GQ009750; GQ009436] from the human skin microbiome. *Caedibacter caryophilus* ^a^ (T); 221. [X71837].	99.0 94.2	KF983493
	2 % AP-K43	Uncultured bacterium clones 16S ribosomal RNA gene, partial sequence [GQ009750; GQ009436] from the human skin microbiome. *Caedibacter caryophilus* ^a^ (T); 221. [X71837].	94.0 90.8	KF983494
*Proteobacteria* (Gamma-class) (90 %)	22.4 % AP-K2	Uncultured Legionellales bacterium clone Hv(lab)_1.9 16S ribosomal RNA gene, partial sequence [EF667907] microbiota in the basal metazoan *Hydra*. *Legionella pneumophila* ^a^ (T); Philadelphia 1 [AE017354].	98.0 96.0	KF983495
	12.4 % AP-K48	Uncultured Legionellales bacterium clone Hv(lab)_1.9 16S ribosomal RNA gene, partial sequence [EF667907] microbiota in the basal metazoan Hydra. *Legionella pneumophila* ^a^ (T); Philadelphia 1 [AE017354].	98.0 96.0	KF983496
	6.2 % AP-K4	Uncultured bacterium clones 16S ribosomal RNA gene, partial sequence [JF681650, JF681408] from the Gastrointestinal tract of a wood-eating fish. *Legionella rowbothamii* ^a^ (T); LLAP6 [X97359].	96.0 91.4	KF983497
	28.6 % AP-K5	Uncultured gammaproteobacterium clone 61-01-00d090 small subunit ribosomal RNA gene, partial sequence [DQ316803] in uranium-contaminated subsurface sediments. *Methylococcus capsulatus* ^a^ (T); Texas = NCIMB 11853 [AJ563935].	95.0 91.2	KF983498
	2 % AP-K7	Uncultured gamma proteobacterium clone 61-01-00d090 small subunit ribosomal RNA gene, partial sequence [DQ316803] in uranium-contaminated subsurface sediments. *Natronocella acetinitrilica* ^a^ (T); ANL 6–2 [EF103128].	95.0 91.2	KF983499
	2 % AP-K19	Uncultured gamma proteobacterium clone 61-01-00d090 small subunit ribosomal RNA gene, partial sequence [DQ316803] in uranium-contaminated subsurface sediments. *Natronocella acetinitrilica* ^a^ (T); ANL 6–2 [EF103128].	95.0 91.2	KF983500
	8.2 % AP-K10	Uncultured bacterium clone ncd266e08c1 16S ribosomal RNA gene, partial sequence [HM270496] skin microbiome associated with disease flares and treatment in children with atopic dermatitis. *Perlucidibaca piscinae* ^a^ (T); IMCC1704. [DQ664237].	99.0 96.2	KF983501
	8.2 % AP-K50	*Acinetobacter* spp. 16S ribosomal RNA gene, partial sequence [KJ806334, AB859733, JX490072, JX290088, and JQ618289]. *Acinetobacter lwoffii* ^a^ (T); DSM 2403 [X81665].	99.0 99.3	KF983502
*Actinobacteria* (2 %)	2 % AP-K44	*Nocardiopsis salina* strain YIM 90010 16S ribosomal RNA, partial sequence [NR_025768, AY373031] a novel halophilic actinomycete isolated from saline soil in China. *Nocardiopsis salina* ^a^ (T); YIM 90010. [AY373031].	99.0 99.8	KF983503
Nonclassified (2 %)	2 % AP-K47	Environmental 16 s rDNA sequence from Evry wastewater treatment plant anoxic basin [CU466697] microbial community composition of an anoxic basin of a municipal wastewater treatment plant. *Vampirovibrio chlorellavorus* ^a^ (T); ICPB 3707 [HM038000].	99.0 85.7	KF983504

^a^Validated species as closest relatives retrieved from the RDPII database

Sequences analyses revealed the dominance of the phylum *Proteobacteria*, which accounted for 96 % of the screened clones (Table 1), and consisted of members of the classes *Alpha-* (6 % of the screened clones) and *Gammaproteobacteria* (90 %). The *Alphaproteobacteria* (clones AP-K3, AP-K32, and AP-K43) showed to be most related (99–94 % similarity) to uncultured bacterial clones from the human skin microbiome [29] and, with a lower similarity (94.4–90.8 %), to the cultured *Caedibacter caryophilus* [30]. Bacteria of the genus *Caedibacter* are symbionts, which inhabit cell compartments of several *Paramecium* species.

The *Gammaproteobacteria* were represented by sequences most related (98 % similarity) to an uncultured Legionellales bacterium clone (34.8 % of the screened clones, represented by clones AP-K2 and AP-K48), detected in the microbiota associated with the phylogenetically ancient epithelia of the basal metazoan *Hydra* [31] and, with a lower similarity (96 %), to the cultured *Legionella pneumophila. L. pneumophila* is a human opportunistic pathogen and has the ability to survive in protozoa, mammalian macrophages, and inhospitable environmental niches [32]. Another 6.2 % of the sequences (represented by clone AP-K4) were most affiliated (96 % similarity) with uncultured bacterial clones found in the gastrointestinal tract of the wood-eating fish *Panaque nigrolineatus* [33], but these sequences were also related, although with a lower similarity (91.4 %), to *Legionella rowbothamii* [34]. 32.6 % of the sequences (represented by clones AP-K5, AP-K7, and AP-K19) were shown to be most affiliated (95 % similarity) with an uncultured gammaproteobacterium clone detected in radionuclide-contaminated subsurface sediments [35]. However, when using the RDPII database, 28.6 % of the sequences (clone AP-K5) affiliated with a lower similarity (91.2 %) with *Methylococcus capsulatus* [36], and 4 % of the sequences (clones AP-K5 and AP-K7) affiliated with a lower similarity (91.2 %) with *Natronocella acetinitrilica. N. acetinitrilica* belongs to moderately salt-tolerant obligate alkaliphiles, which are pigmented due to a high concentration of carotenoids in the cells, dominated by zeaxanthin [37]. 8.2 % of the *Gammaproteobacteria* (represented by clone AP-K10) were related to an uncultured bacterium from the skin microbiome associated with disease flares and treatment in children with atopic dermatitis [38] and, with a lower similarity (96.2 %), to *Perlucidibaca piscinae*, a freshwater bacterium that belongs to the family *Moraxellaceae* [39]. The rest of the *Gammaproteobacteria* (8.2 %) were shown to be affiliated with cultured species of the genus *Acinetobacter*. Species of this genus were previously detected by molecular methods on other parchment documents [40, 41].

The phylum *Actinobacteria* represented 2 % of the screened clones and the sequences were related to halophilic species of the genus *Nocardiopsis*, as *Nocardiopsis salina* isolated from saline soil [42]. Finally, 2 % of the screened clones affiliated with 16S rDNA sequences retrieved from the microbial community composition of an anoxic basin of a municipal wastewater treatment plant [43]. These last sequences yielded a very low similarity when compared to sequences of the RDPII database (85.7 %), being associated to *Vampirovibrio chlorellavorus* and member of the *Deltaproteobacteria.* However, due to the very low similarity with the validated species, it was not possible to properly classify these sequences (see Table 1).

In summary, the microbiota inhabiting this parchment manuscript was dominated by uncultured members of the *Gammaproteobacteria*, many of them related to sequences of endosymbionts. However, the presence of sequences most related to microorganisms from the human skin microbiome [29], such as the uncultured clones AP-K3, AP-K32, AP-K43, and AP-K10 detected in this study, is noteworthy. Sequences most related to the human skin microbiome have been recently found on parchment samples showing a phenomenon of purple stains deterioration [40] and have been also related to the deterioration of the skin of mummies [7]. In addition, and interestingly, results also showed the presence of sequences related to halophilic species of the genera *Nocardiopsis* and *Natronocella* [37, 42]. Halophilic bacteria were also dominant among the microbiota detected in biodeteriorated parchment samples recently investigated [40].

#### Fungal Community

Molecular analyses revealed differences among the fungal communities colonizing three different areas of one folio of the Archimedes Palimpsest that were sampled with cotton swabs (Fig. 3b–d, Table 2). Sample AP1 (location B in Fig. 1b), taken from a stained and degraded area, was found to be dominated by *Mucor* spp. (93.4 %), namely *Mucor hiemalis*, a fungal plant pathogen of which some of the related sequences were recovered from decaying bioenergy plants [44]. The rest of the sequences of sample AP1 affiliated with members of the Ascomycetes, 2.2 % of sequences with *Penicillium* spp., namely with species isolated from cork samples [45], and another 2.2 % of sequences with an uncultured fungal clone detected in continental and marine air and, with a lower similarity (98 %), with *Phaeosphaeria* spp. Finally, 2.2 % of sequences were affiliated with basidiomycetous yeasts, namely with *Trichosporon inkin* [46].

**Table 2:** Phylogenetic affinities of the fungal ITS coding sequences retrieved from swab samples of the Archimedes Palimpsest

Phylum	Clones (%) and selected clone	Closest identified phylogenetic relatives (EMBL accession numbers)	Similarity (%)	Acc. Nr.
Sample AP1				
*Zygomycota*	82.6 % AP1-F1	*Mucor* spp. [HQ630989, EU326196, AY243949].	100	KF983505
	4.3 % AP1-F6	*Mucor* spp. [HQ630989, EU326196, AY243949].	99.0	KF983506
	4.3 % AP1-F46	*Mucor* spp. [HQ630989, EU326196, AY243949].	99.0	KF983507
	2.2 % AP1-F49	*Mucor* spp. [HQ630989, EU326196, AY243949].	99.0	KF983508
*Ascomycota*	2.2 % AP1-F24	*Penicillium* spp. [GU372906, GU372905] isolates from cork samples.	99.0	KF983509
	2.2 % AP1-F13	Uncultured fungal clone [GU053879]. *Phaeosphaeria* spp. [U77359, GQ922523].	99.0 98.0	KF983510
*Basidiomycota*	2.2 % AP1-F10	*Trichosporon inkin* [JX463242, NR_073243].	99.0	KF983511
Sample AP2				
*Ascomycota*	86.4 % AP2-F2	Uncultured fungus clone F1-O15 [JX984691, GU054220, FJ820820] from air. *Blumeria graminis* strains [AB273546, AB273543, AB273542] a powdery mildew fungus of cereals.	99.0 99.0	KF983512
	4.5 % AP2-F4	Uncultured fungus clone F1-O15 [JX984691, GU054220, FJ820820] from air. *Blumeria graminis* strains [AB273546, AB273543, AB273542] a powdery mildew fungus of cereals.	99.0 99.0	KF983513
	9.1 % AP2-F45	*Penicillium oxalicum* [KF152942], lignocellulolytic enzymes production by a *Penicillium*. *Penicillium* sp. 3 BRO-2013 [KF367495], fungi in water source and their potential pathogenicity.	99.0 99.0	KF983514
Sample AP3				
*Ascomycota*	23.7 % AP3-F1	Uncultured fungus clone 20–52 [KC884473], the diversity of fungal community in permafrost soil. *Cladosporium* sp. Strains [KF367544; KF367490], occurrence of fungi in water sources and their potential pathogenicity. *Cladosporium cladosporioides* strain ML370 [KC692219].	100 100 100	KF983515
	2.6 % AP3-F3	Uncultured fungus clone 20–52 [KC884473] Diversity of fungal community in permafrost soil. *Cladosporium* sp. Strains [KF367544; KF367490], occurrence of fungi in water sources and their potential pathogenicity. *Cladosporium cladosporioides* strain ML370 [KC692219].	99.0 99.0 99.0	KF983516
	18.4 % AP3-F7	Uncultured fungus clone 20–52 [KC884473], the diversity of fungal community in permafrost soil. *Cladosporium* sp. Strains [KF367544; KF367490], occurrence of fungi in water sources and their potential pathogenicity. *Cladosporium cladosporioides* strain ML370 [KC692219].	100 100 100	KF983517
	13.2 % AP3-F16	Uncultured fungus clone 20–52 [KC884473], the diversity of fungal community in permafrost soil. *Cladosporium* sp. Strains [KF367544; KF367490], occurrence of fungi in water sources and their potential pathogenicity. *Cladosporium cladosporioides* strain ML370 [KC692219].	99.0 99.0 99.0	KF983518
	2.6 % AP3-F20	Uncultured fungus clone [JX123348, JX123346], fungal endophytes. *Aspergillus* spp. [JQ717355, HE608807, HQ832961].	99.0 99.0	KF983519
	2.6 % AP3-F4	*Acremonium charticola* culture-collection UOA/HCPF < GRC>:14413 [KC253940], common and emerging mold pathogens in Greece.	98.0	KF983520
	5.3 % AP3-F5	*Hypoxylon* spp. [GU166476, JN198512].	88.0	KF983521
	2.6 % AP3-F32	Uncultured *Pezizomycotina* clones [JF449770, JF449825] from leaf litter.	98.0	KF983522
	2.6 % AP3-F44	*Stachybotrys* sp. [JN093263] from decaying hardwood. *Stachybotrys longispora* [AF081482] toxigenic fungal species of *Stachybotrys.*	98.0 98.0	KF983523
	2.6 % AP3-F6	*Knufia petricola* [AJ507323], lithobiontic dimorphous dematiaceous fungus isolated from marble rock.	91.0	KF983524
	2.6 % AP3-F40	*Knufia petricola* [AJ507323], lithobiontic dimorphous dematiaceous fungus isolated from marble rock.	99.0	KF983525
	18.5 % AP3-F11	Uncultured *Coniosporium* clone MP45 [HM136653] from plant roots. *Coniosporium* sp. MA4666 [AJ971447], microcolonial fungi from antique marbles in Perge/Side/Termessos (Antalya/Turkey).	99.0 98.0	KF983526
	2.6 % AP3-F27	Uncultured fungal clones [JX984717, JX984762] atmospheric fungal communities. *Capnobotryella* spp. [AJ972860, AJ972854], microcolonial fungi from antique marbles in Perge/Side/Termessos (Antalya/Turkey).	98.0 98.0	KF983527

Swab sample AP2 (location C in Fig. 1b), taken from a healthy area with no visible staining or degradation, was shown to be dominated (90.9 %) by sequences related to an uncultured fungus clone from air [47] and to the cultivated *Blumeria graminis*, a powdery mildew fungus of cereals [48]. The rest of the sequences (9.1 %) were related to *Penicillium* spp., possessing lignocellulolytic enzymes, and to *Penicillium* spp. from water sources and possessing potential pathogenicity [49]. Members of the genera *Mucor* and *Penicillium* have previously been isolated from samples of old damaged parchment and tested for cellulolytic and proteolytic activities. Isolates belonging to both genera showed a marked proteolytic activity [50]. *Penicillium* species were also dominant members of the fungal communities colonizing other deteriorated parchment samples [40].

Swab sample AP3 (location A in Fig. 1b), taken from an intensely stained and degraded area, was found to have the highest fungal diversity. Of the sequences, 57.9 % were related to an uncultured fungus clone from soil and to cultivated species of *Cladosporium* isolated from water sources and possessing potential pathogenicity [49]. Affiliated with species of the genus *Aspergillus* and another 2.6 % with *Acremonium charticola*, a common and emerging mold pathogen in Greece (nonpublished), were 2.6 % of the sequences. Members of the genus *Aspergillus* [40, 51–53] and *Acremonium* [52] have been described as active biodeteriogen agents on ancient parchments. In addition, 5.3 % of the sequences were related to *Hypoxylon* spp., from endophytic fungal communities of *Taxus chinensis* var. *mairei* [54], 2.6 % of sequences to uncultured Pezizomycotina clones from leaf litter and other 2.6 % to *Stachybotrys* spp. from decaying hardwood and *S. longispora*, a toxigenic fungal species of *Stachybotrys* [55]. Surprisingly, the remaining sequences were found to be related to sequences of rock-inhabiting fungi that are frequently isolated from marble and lime stones, as *Knufia petricola* (5.2 % of sequences), *Coniosporium* spp. (18.5 %), and *Capnobotryella* spp. (2.6 %) [56].

Finally, it is worth to mention that in all three swab samples, around 2 % of the sequenced clones showed to be affiliated with DNA of plants, represented in Fig. 3b–d as lane P. The ITS primers ITS1 and TS4, even if widely used for the specific amplification of fungi, are known to amplify plant ITS regions as well [18]. These sequences were discarded and not taken into consideration for the statistical analyses of the fungal clones showed on Table 2.

### Quantitative Real-Time PCR Analyses of the β-Actin Gene

In a recent study, the quantification of the β-actin gene by quantitative real-time PCR (qPCR) has been used as an indicator of fungal abundance in cultural heritage materials including parchment [57]. Following the established protocol described by Ettenauer et al. [57], the quantitative real-time PCR allowed the detection of the fungal abundance in all three sampled areas of the parchment (Table 3). The detected β-actin copies were referred to the total amount of DNA extracted from each parchment sample. The resulting values revealed that the copies of β-actin gene were 1–2 decimal power higher in damaged areas than in the healthy area, indicating an increased abundance of fungal colonization on those areas of the manuscript (Table 3).

**Table 3:** Quantification of β-actin on the different sampled areas of the Archimedes Palimpsest by qPCR

Sample	Copies/ng extracted DNA
AP1-mold-stained	32.36
AP2-healthy	9.08
AP3-mold-stained	132.22

The structure of actin genes can be examined across broad evolutionary distances because actin is highly conserved and is ubiquitous in eukaryotes. Actin is encoded by a multigene family in all animals, protozoa, and plants so far examined, but it has a tendency to be encoded by a single gene in fungi [58–60]. Therefore, the qPCR method used in this study offers a simple, rapid, and reliable tool for the precise quantification of fungal cells. This is not the case when using universal rRNA primers, due to the great variation of the number of rRNA gene clusters in a genome and in a species that makes it difficult to estimate the number of fungal individuals [61].

Considering the differences obtained from the three swab samples, in terms of fungal species and abundance, the fungal contamination on the manuscript possibly comes from an airborne contamination. In indoor environments, surface dust sampling can serve as a possible historical marker for cumulative exposures [62]. Moreover, dust deposition in indoor environments is quantitatively influenced by green areas and the total area of vegetation in the vicinity of the sampled location [63]. In theory, the sampled folios bear the DNA of all the microorganisms that have come and gone in a very long succession of events along the history of the object, although the number of samples that could be taken and used in the survey are statistically not sufficient to tell us something about the past events to which the manuscript was exposed. The fungal component of dust biodiversity is greatly underestimated, either because only a few studies have provided thorough mycological characterizations of indoor dust [64] or because some of the fungal species that colonize indoor environments are easily overlooked due to their peculiar growth requirements. Apart from the natural variability in the number of different fungal and bacterial species that can be found on the pages of a book, there is also variability in the statistics representing their numbers, depending on the sampling methods employed and the peculiarities of the material investigated. According to some authors [65], fungi that have as a growth requirement water activity (aw) above 0.85 can be regarded as indicator organisms for the presence of damp conditions in indoor environments. A survey carried out by Salonen et al. [66] demonstrated that *Cladosporium* isolates were the most common fungi detected in the samples collected from indoor air and in settled dust, in both mold-afflicted and “healthy” control buildings. According to different authors [65, 66], the airborne concentrations of *Penicillium*, *Aspergillus versicolor*, and yeasts can be considered good indicators of mold-related problems in indoor environments. The fungal records associated with the damaged folios of the Archimedes Palimpsest can be partly due to the airborne dispersion of spores from other sources, but some species can actually be parchment colonizers or secondary colonizers that grew on the already damaged collagen fibers. In the early 1970s, Gallo and Strzelczyk [67] repeatedly isolated *Aspergillus*, *Penicillium*, and *Mucor* strains from damaged parchments.

### SEM Observations

**Fig. 4:**
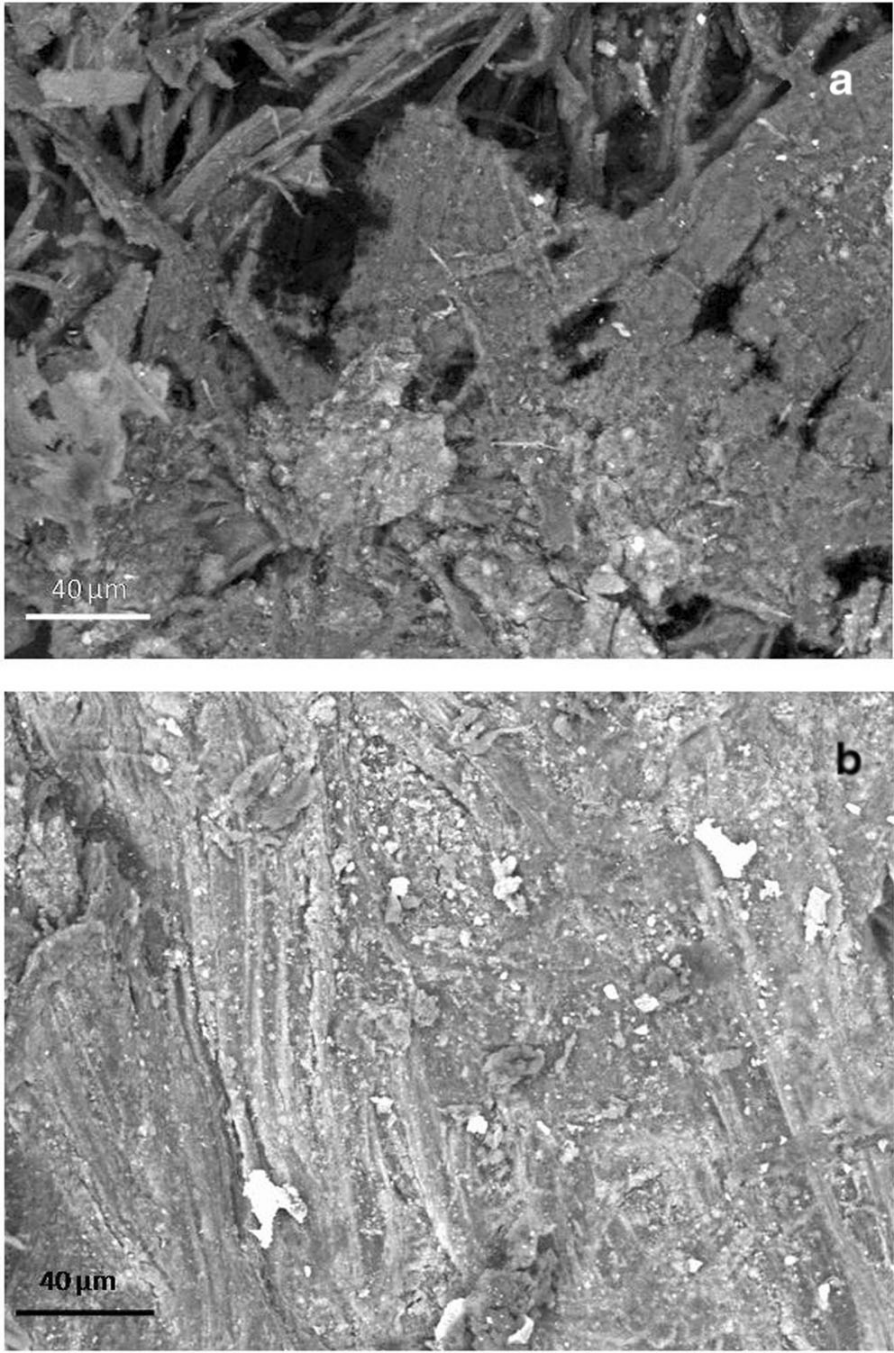
Surface of the core samples taken from a stained/degraded area of the parchment (**a**) and the healthy/control area of folio 68v (**b**). The SEM images were obtained at variable pressure (50 Pa) with a backscattered electron detector on uncoated material; scale, 40 μm. In **a**, a general breakdown of the parchment is visible, while in **b** the surface is intact with a uniform layer of mineral material that appears brighter in respect to the collagen fibers because of its higher atomic number. Both images were taken on the flesh side of each sample (for chemical comparison, see Table 4, sample; Healthy flesh: H_f, versus Damaged_flesh: D_f)

**Fig. 5:**
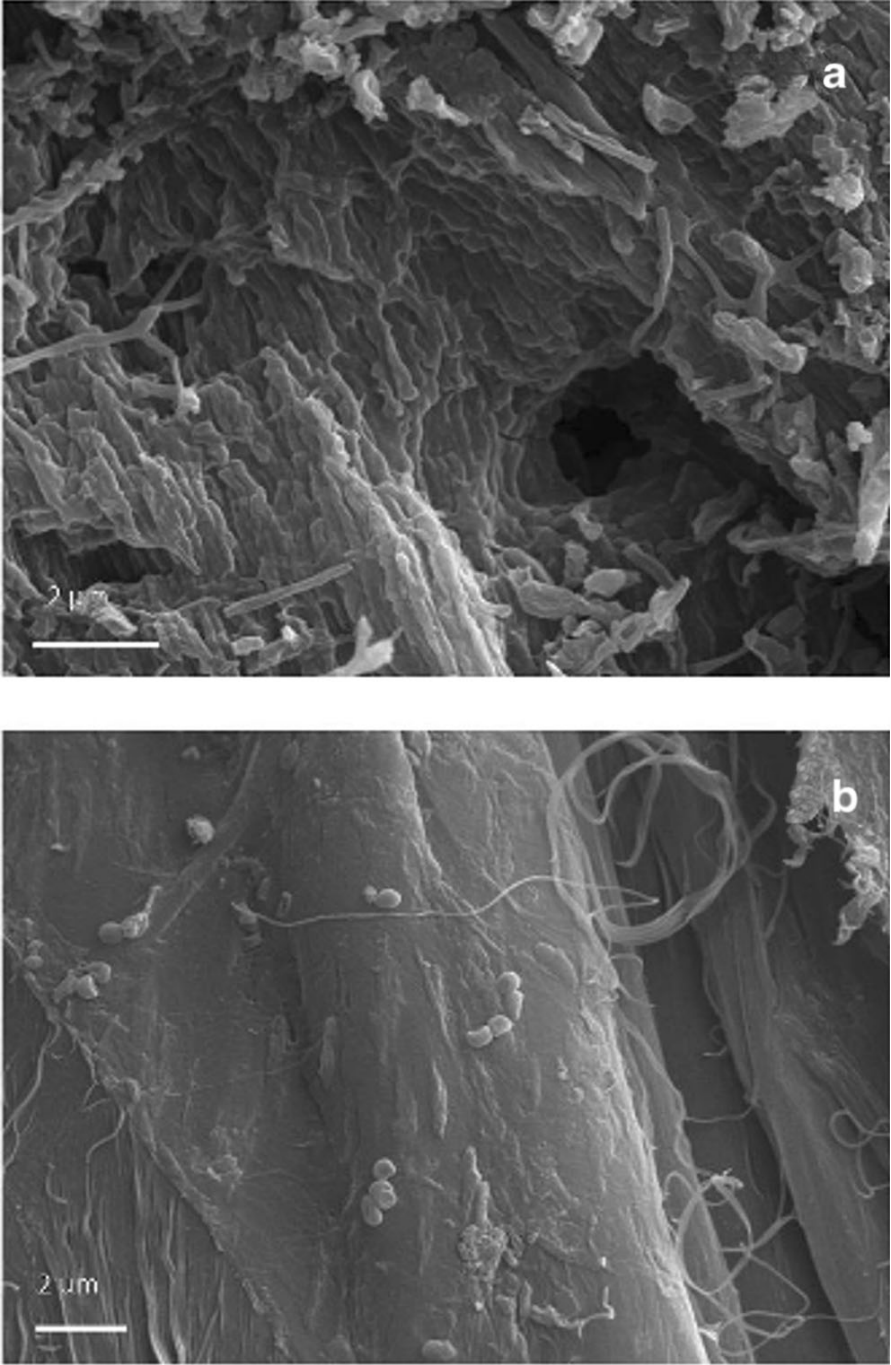
Samples taken from a stained/degraded area (**a**) and a healthy/control area of folio 68v (**b**). The pictures were obtained with high-vacuum secondary electron SEM imaging on gold-sputtered core samples; scale, 2 μm. A profound structural damage consisting of holes, cracks, and fissures was documented by HV-SEM imaging on both the flesh and hair sides of the first sample (**a**). **b** Shows a collagen fiber from the healthy parchment core sample; the surface is smooth and compact, although a few bacterial cells, appearing in chains, were observed adhering to the surface of the fiber

**Fig. 6:**
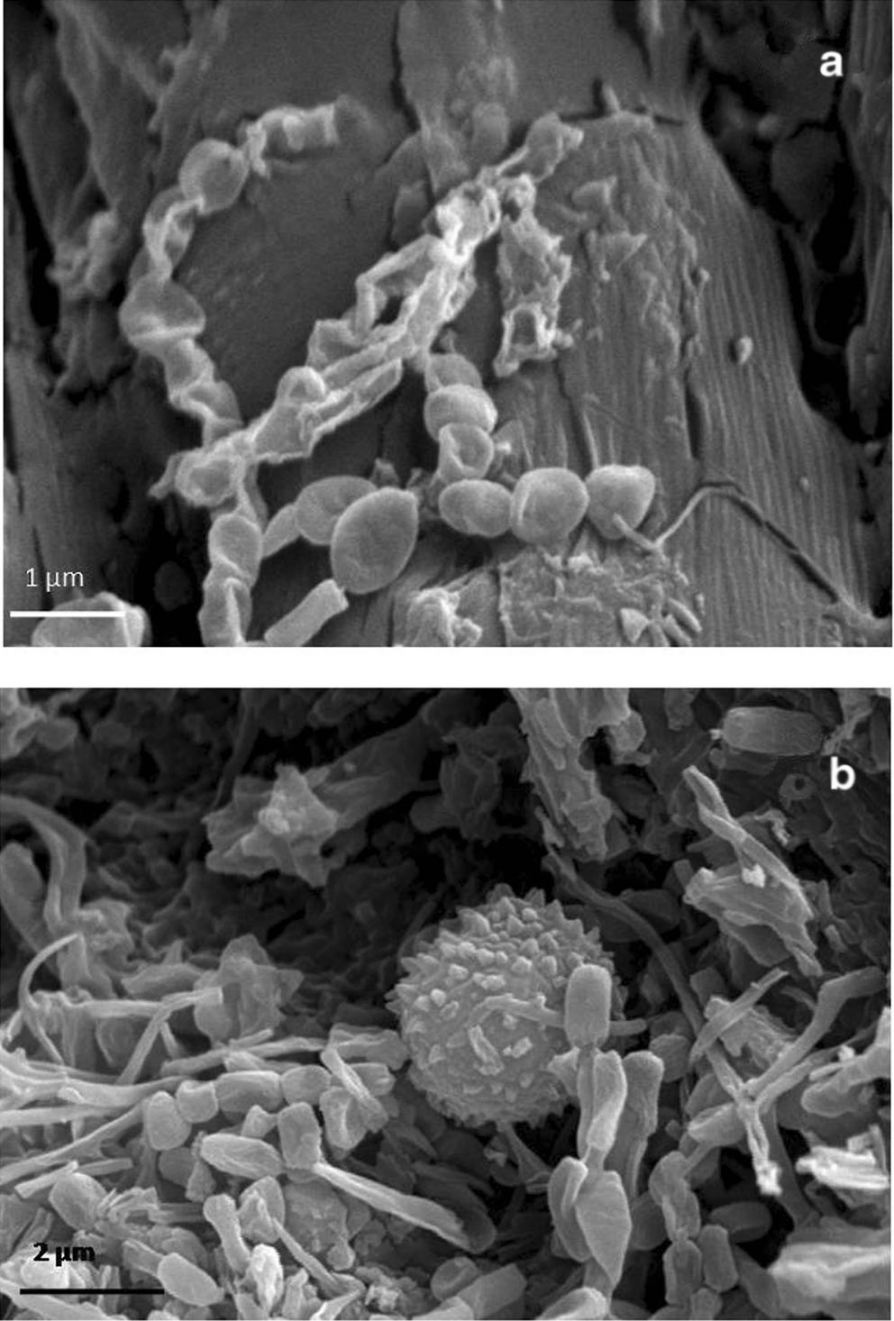
HV-SEM images of gold-sputtered core samples of parchment taken from folio 68v. **a** Taken from the core sample corresponding to the “healthy” area; it shows the bacterial structure at the initial formation of a spore chain. It represents an initial attack to the collagen fiber, which is still visible and integer on the backward; bacterial cells (less than 1 μm in diameter) form chains that are morphologically consistent with Actinomycetales taxon; the filaments appear branched and are fragmenting into spores; scale, 1 μm. **b** Taken from the core sample corresponding to the “purple/damaged” area; it shows chains of bacterial spores and filaments and a single fungal conidia, globose, and with echinated ornamentation; the collagen fiber is no longer distinguishable; scale, 2 μm

**Fig. 7:**
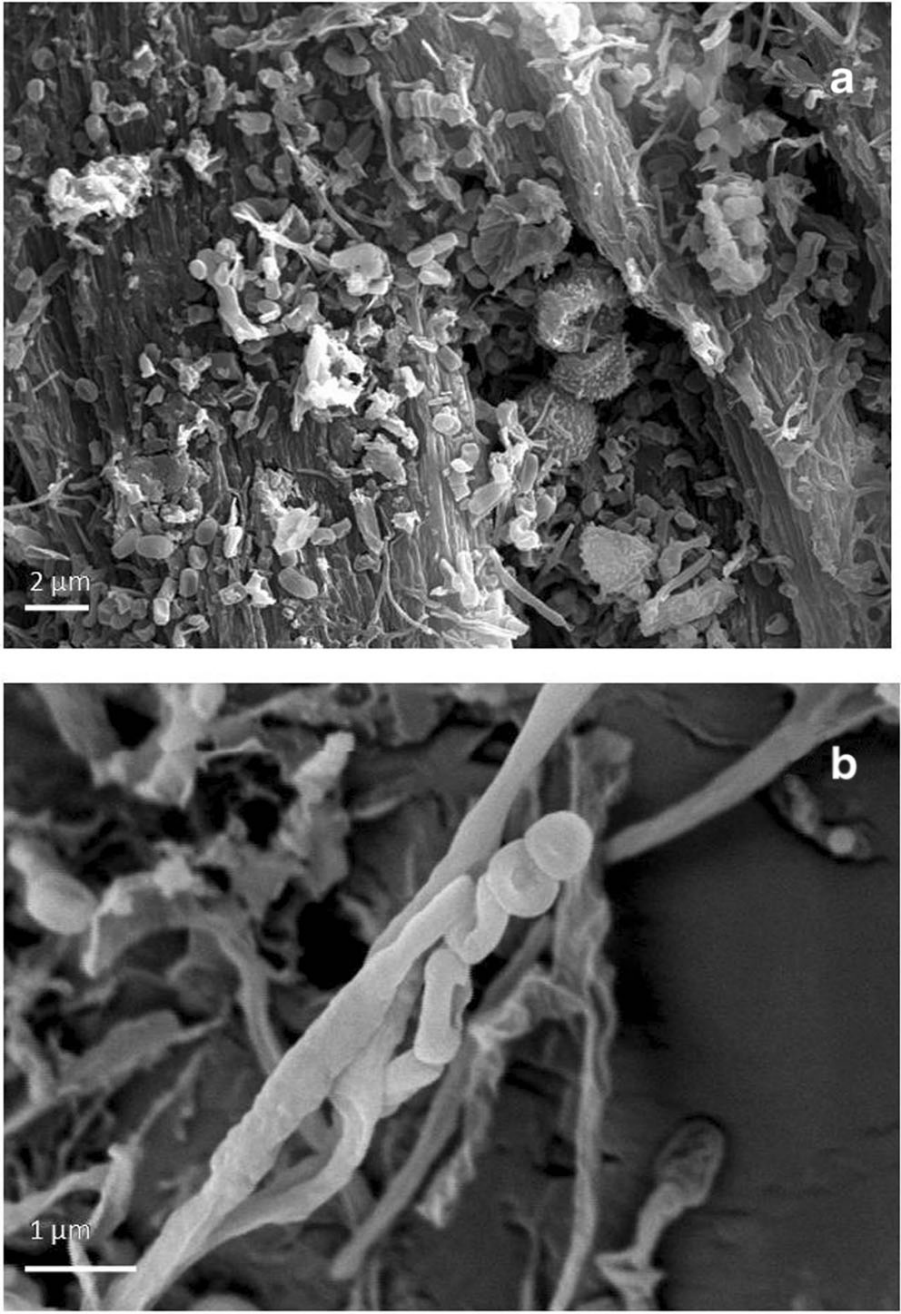
HV-SEM images of gold-sputtered core samples of stained/degraded parchment from two different folios. **a** Chains of bacterial spores and filaments and a chain of fungal conidia, globose, and with echinated ornamentation; the fungal conidia appear covered with bacterial filaments. In the background, the degraded collagen fibers are also visible; *scale* 2 μm. **b** Aerial bacterial filaments appear coarsely wrinkled and branched, fragmenting into spores; scale, 1 μm

VP-BSD SEM imaging at low magnification showed a different pattern of salts distribution on the parchment surface in core samples taken from damaged and healthy areas; salts dislocation occurred more frequently on the damaged surface than the healthy one (Fig. 4a, b). The appearance of the stained and degraded samples indicated that the microbial attack was associated with the loss or consumption of the outer layers of the parchment. The lyses of collagen fibers and a profound structural damage was documented by HV-SEM imaging on both the flesh and hair sides of the core sample taken from the purple stained areas of the folio. This showed that the organism that caused the damage was actually using collagen as carbon source (Fig. 5a, b). The observation of gold coated samples by means of SEM in high vacuum mode showed the presence of both bacterial and fungal cells between the collagen fibers (Figs. 6 and 7). In these images, a huge number of bacterial cells were observed, compared to only a few echinated fungal conidia that were present (Figs. 6b and 7a). Bacterial cells (less than 1 μm in diameter) documented by SEM form chains that are morphologically consistent with the genus *Nocardiopsis*, which was also identified by molecular methods. *Nocardiopsis* was described by Meyer [68] and is a genus of aerobic actinomycetes that includes several species [69]. It includes moderately halophilic species capable of producing alkaline proteases [70]; some species also produce red to deep orange pigments [71]. A detailed picture of the prevailing morphology of the filamentous bacteria that occur in the manuscript is reported in Fig. 6a. The filaments that appear coarsely wrinkled are often branched and fragmenting into spores (Fig. 7a, b). Aerial filaments are well developed and abundant. Some chains of bacterial cells were also found adhered to intact collagen fibers as showed in Fig. 5b. The three sample cores of parchment (healthy and damaged) were very small when compared to the folios of the manuscript and therefore not very representative of the whole object. Despite the difficulties presented by the uniqueness of the object, its value and the ethical and practical problems linked to sampling, it became quite clear from the SEM images, that the damage is linked to an attack by an apparently unique species of filamentous bacteria rather than to the presence of filamentous fungi. The few fungal cells that were found in SEM images taken of the entire surface of the available sample appeared in chains, echinated, and covered with bacterial filaments (Figs. 6b and 7a). This suggests that (a) the fungi present on the damaged samples may correspond (according to shape, ornamentation, and dimension), among those found with molecular techniques, to a *Penicillium* or an *Aspergillus* species and (b) the fungi grew on the parchment before or together with the collagenolytic bacterium.

### EDX Results

Table 4 reports the average values of chemical elements present on the surface of the three core samples taken from stained and degraded and healthy, intact areas. EDS is a not quantitative analytical method, due to the non-uniform density of the material (parchment), yet a comparison performed on a statistically substantial number of measurements, conducted through a careful standardization of the instrumental parameters may still have a value for the description of the samples. Nitrogen cannot be measured like the other elements due to matrix effect, which makes it impossible to construct a calibration curve that correlates the signal directly with the nitrogen content for any type of sample [72]. However, following Gazulla et al. [73], a rough estimation of the N content of the samples is reported in Table 4.

**Table 4:** Microanalysis (EDX) results. Comparison of elemental composition of parchment samples. Data are reported as weight %. One-way ANOVA/Tukey’s *t* test comparisons are reported; statistically significant differences (*p* < 0.001) in elemental composition is marked with different letters (A, B, C). Mean values of 60 measurements ± standard deviation are reported

Element	Sample	Mean	Standard deviation	Group
Al	D_f	0.06	0.10	B
Al	D_h	0.35	0.41	B
Al	H_f	0.25	0.24	B
Al	H_h	1.38	0.60	A
C	D_f	56.06	3.14	A
C	D_h	57.93	5.03	A
C	H_f	44.67	3.09	B
C	H_h	42.47	5.98	B
Ca	D_f	1.79	0.53	B
Ca	D_h	2.74	1.23	B
Ca	H_f	8.61	3.60	A
Ca	H_h	4.02	2.37	B
Cl	D_f	1.20	0.33	A
Cl	D_h	1.06	0.43	A
Cl	H_f	1.04	0.19	A
Cl	H_h	0.80	0.56	A
Fe	D_f	0.00	0.00	C
Fe	D_h	0.20	0.36	BC
Fe	H_f	0.51	0.58	AB
Fe	H_h	0.69	0.31	A
K	D_f	0.31	0.09	C
K	D_h	0.63	0.21	B
K	H_f	0.48	0.13	BC
K	H_h	0.82	0.27	A
Mg	D_f	0.08	0.11	B
Mg	D_h	0.17	0.16	B
Mg	H_f	0.10	0.15	B
Mg	H_h	0.37	0.24	A
N	D_f	17.89	2.85	A
N	D_h	14.23	3.25	BC
N	H_f	16.51	1.90	AB
N	H_h	12.65	2.09	C
Na	D_f	0.42	0.19	AB
Na	D_h	0.55	0.08	A
Na	H_f	0.15	0.21	C
Na	H_h	0.41	0.21	B
O	D_f	21.54	2.25	BC
O	D_h	20.65	3.49	C
O	H_f	25.76	1.55	B
O	H_h	31.92	5.36	A
P	D_f	0.00	0.00	C
P	D_h	0.18	0.19	C
P	H_f	0.75	0.28	A
P	H_h	0.40	0.21	B
S	D_f	0.40	0.10	A
S	D_h	0.56	0.18	A
S	H_f	0.43	0.10	A
S	H_h	0.42	0.34	A
Si	D_f	0.24	0.17	B
Si	D_h	0.73	0.71	B
Si	H_f	0.74	0.29	B
Si	H_h	3.64	2.47	A

*H_h* healthy hair side, *H_f* healthy flesh side, *D_h* damaged hair side, *D_f* damaged flesh side

Both sides (flesh and hair) of each core sample showed the presence of the following elements: Na, Mg, Al, Si, P, S, Cl, K, Ca, and Fe. The element that was more abundant, apart from C, H, N, and O, was Ca; it was found to be significantly higher in the healthy sample (H) and specifically on the flesh side. The healthy sample was also rich in Si, Al, Mg, and P. Na and Cl were more present on both sides of the damaged sample (D). Traces of Fe were found mainly in the healthy sample.

The elements Mg and K were also present in all the samples and these are a result of manufacturing processes, in particular from chlorides that were used as preservatives in the drying of the freshly flayed skins before they were made into parchment. Mg and K, together with Fe can also originate from the hide itself, since they occur naturally at low concentrations in leather and parchment [74].

**Fig. 8:**
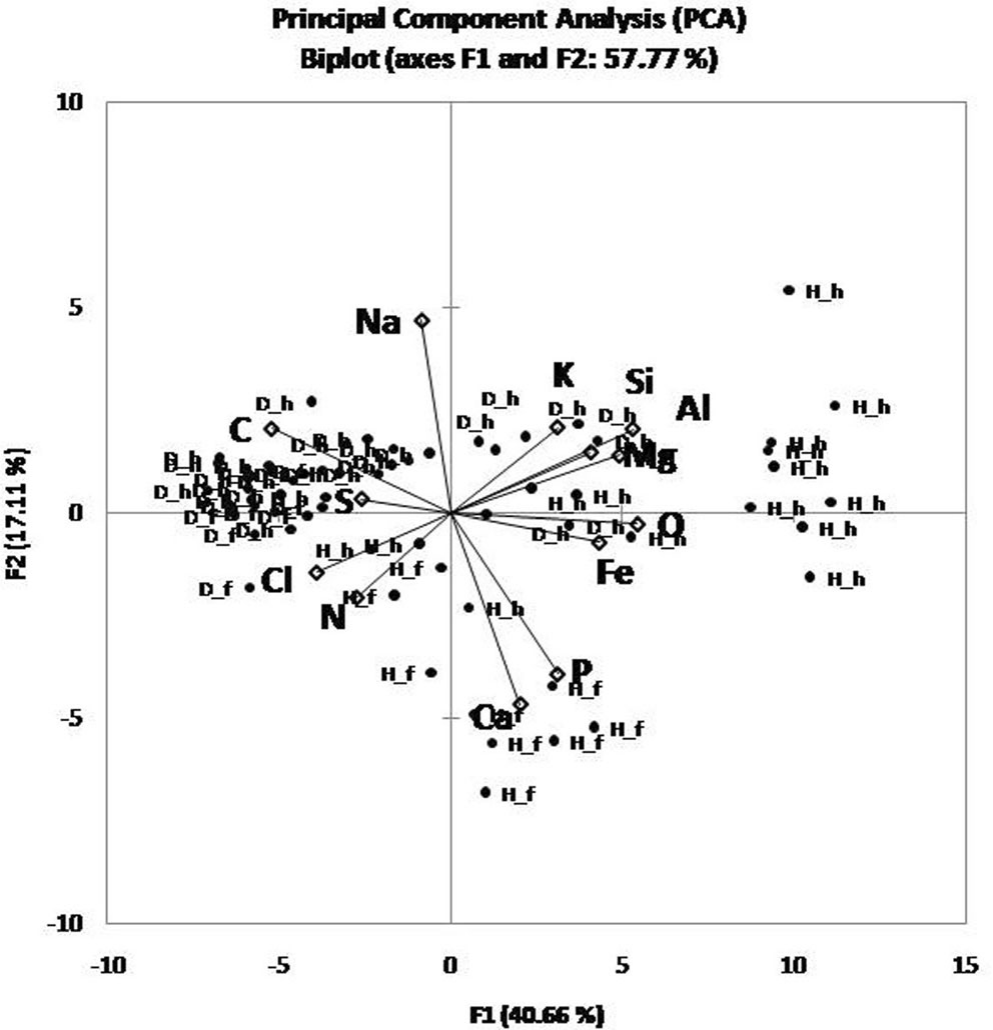
PCA analysis plot of the first two components (57.77 % of the total variation). The variables used were the chemical elements obtained from the parchment core samples by means of EDS analysis (C, N, O, Ca, Si, Al, Cl, Na, S, P, Mg, and Fe). *H_h* healthy hair side, *H_f* healthy flesh side, *D_h* damaged hair side*, D_f* damaged flesh side

The PCA (Fig. 8) where the first two components accounted for the 57.77 % of the variability (and the cumulated first four components accounted for 77 % of the variability, which is data not reported in the plot) showed a clear separation between damaged and healthy samples along the first PC, but also a grouping of data taken from the hair side and the flesh side of the core samples (second PC). The PCA plot indicates that some chemical elements present in the samples co-exist as a consequence of manufacturing processes. This is the case with Si and Al, and Mg and K, which are present due to the use of pumice stone (composed of silicates and oxides) for smoothing the parchment surface in preparation for writing. These elements were mainly correlated with the healthy parchment samples (H_f, H_h) whose observations are concentrated on the right side of the plot. On the left side of the PCA plot (Fig. 8), there is a correlation between the elements Na and Cl and the damaged samples (D_f, D_h). Salting (mainly with marine salt) was undertaken during the first phase of parchment manufacture. In particular, salts (mainly sodium or potassium chlorides, ammonium chloride or sulfate) were used at the beginning of the process to inhibit microbial activity and prevent the raw animal hides from putrefaction [1] before they were made into parchment. Perhaps it is not a coincidence that the halophilic and proteolytic bacteria that usually attack parchment are typical of coastal marine environments. For example *N. salina*, which was identified both by molecular methods and more roughly by its morphology with SEM, is a species associated with saline soils and marine salt [42].

According to the PCA plot, the separation between the hair and flesh sides of the parchment samples is more pronounced in the healthy sample, while in the purple-stained and degraded samples, almost no separation could be seen. The ANOVA data showed that, on average, the healthy sample contained more inorganic elements, as confirmed by the SEM BSD imaging (Figs. 2 and 4). This is apparently due to a detachment of the outer layers of parchment and a leaching of the inorganic elements in the stained and degraded samples as a result of microbial activity.

## Conclusions

The combined approach used in this study, using molecular and microscopic techniques, provides an insight into the microbiota associated with the biodeterioration phenomenon observed on the Archimedes Palimpsest, an unusual medieval manuscript made of parchment. The observation of gold-coated samples by means of SEM in high vacuum mode showed the presence of both bacterial and fungal cells in the stained and degraded areas, and also in a more limited way in some healthy areas. VP-BSD SEM imaging showed a different pattern of salts distribution on the surface of core samples taken from stained/degraded and healthy areas of two folios of the manuscript. The appearance of the damaged samples indicated that the microbial attack was associated with the loss or consumption of the outer layers of parchment. The lyses of collagen fibers and a profound structural damage could be correlated with the purple stained areas of the folios, showing that the organism that caused the degradation and the stains was actually using the collagen as a carbon source.

Molecular results showed that the bacterial communities present on the three different swab sampled areas on one folio of the manuscript, two of them exhibiting deterioration and one with no visible damage, were identical. The bacterial sequences that were retrieved were most related to sequences associated with the human skin microbiome and to sequences detected in ephitelium, as well as to sequences previously detected on biodeteriorated parchments, suggesting a specific colonization of the Archimedes Palimpsest as well as a potential cause for its deterioration. In particular, *N. salina*, a species associated with saline soils and marine salt, was identified both by molecular methods and more roughly by morphology with SEM. A correlation between the presence of the elements Na and Cl, possibly coming from parchment manufacturing processes, and the purple stains and degradation was also observed.

A great variation was observed among the three swab sampled areas concerning fungal diversity. Samples showing visible damage, such as sample AP1, was dominated by *Mucor* spp. while sample AP3, from a more intensely stained and degraded area of the same folio, showed a much higher fungal diversity with the dominance of *Cladosporium* spp. Sample AP2, taken from a healthy area, was found to be dominated by *Blumeria* spp. SEM imaging showed that some fungi resembling *Penicillium* or *Aspergillus* species grew on the parchment either before or together with the collagenolytic bacterium. Finally, results derived from qPCR analyses directed towards the β-actin gene revealed a higher fungal abundance in the two stained and degraded areas than in the healthy area of the manuscript folio.

Finally, it is important to remark that the molecular techniques used in this study allowed the identification of the ancient and modern microbiota present on the Palimpsests, what is was completely impossible by using classical cultivation techniques in previous surveys, and even enabled an estimation of the abundance of fungi on the damaged and healthy areas. Nevertheless, molecular techniques are developing at a fast pace and new techniques, as high-throughput sequencing by a next-generation sequencer, are evolving for different applications and the ongoing scientific and technological progresses led to metagenomics, transcriptomics, and proteomics, which give a complete overview of the present microorganisms, their activity, and the expressed proteins. The future application of these new techniques for the investigation of objects of cultural heritage will answer many questions that are still open. However, these state-of-the-art methodologies require special instruments, trained personnel, and high costs, and, therefore, only few laboratories are able to perform such studies that, to our knowledge, have not been applied in cultural heritage studies.
